# The role of primary care in improving health equity: report of a workshop held by the WONCA Health Equity Special Interest Group at the 2015 WONCA Europe Conference in Istanbul, Turkey

**DOI:** 10.1186/s12939-016-0415-8

**Published:** 2016-08-05

**Authors:** Ula Jan Chetty, Patrick O’Donnell, David Blane, Sara Willems, World Organization of Family Doctors (WONCA) Special Interest Group on Health Equity

**Affiliations:** 1 General Practice and Primary Care, University of Glasgow, Scotland, UK; 2 Graduate Entry Medical School, University of Limerick, Limerick, Ireland; 3 Department of Family Medicine and Primary Health Care, Ghent University, Ghent, Belgium

**Keywords:** Health equity, Medical education, Primary care, Social determinants of health

## Abstract

The WONCA Special Interest Group on Health Equity was established in 2014 to provide a focus of support, education, research and policy on issues relating to promotion of health equity in primary care settings. In keeping with this remit, the group hosted a workshop at the WONCA Europe conference held in Istanbul in October 2015. The aim of the session was to engage practitioners from across Europe in discussion of the barriers and facilitators to addressing the social determinants of health at practice level and in the training of doctors. This commentary reflects on the main findings from this workshop and how these compare with existing work in this field.

## Background

Despite overall improvements in population health in most countries over the last fifty years, deprivation-related health inequity remains a serious challenge worldwide. The World Health Organization defines the social determinants of health (SDH) as “the circumstances, in which people are born, grow up, live, work and age and the systems put in place to prevent and treat illness” [1]. In order to reduce or prevent health inequities, action is required across all of the social determinants of health including factors such as education, employment and housing. Additionally, health systems have a vital role to play in mitigating the physical and mental health adversity caused by the SDH. However, if good medical care is not readily available and accessible according to the need for it in the population, then health inequities will inevitably widen – reflecting the inverse care law in action [2].

Primary care (General practice/Family medicine) is particularly well placed to support health equity, for a number of reasons. Firstly, it has population coverage, with most patients being registered with a general practitioner (GP) [3]. Secondly, it has direct contact with patients. Again, this varies between countries, but most patients will see their GP as the first point of contact with the health service, and patients are not discharged from their GP, unlike secondary care services [4]. Thirdly, primary care has the potential for continuity, through serial encounters with known healthcare providers. Finally, primary care offers comprehensive coordination of generalist care to patients in their own environment, allowing the doctor to become an ‘expert generalist’ with a good understanding of the social context in which their patients live.

The WONCA Special Interest Group on Health Equity was established in 2014 in response to the Health Equity Workshop held at the WONCA World 2013 conference in Prague. The participants of this workshop expressed the importance of support, education, research and discussion on policy issues relating to promotion of health equity in primary care settings [5]. In keeping with this remit, the WONCA Special Interest Group on Health Equity hosted a second workshop at the WONCA Europe conference held in Istanbul in October 2015. The aim of the session was to engage practitioners from across Europe in discussion of the barriers and facilitators to addressing the SDH at practice level and in the training of doctors. This commentary reflects on the main findings from this workshop and how these compare with existing work in this field.

## Main text

The workshop was attended by General Practitioners and GP trainees from across Europe, including France, Spain, Italy, Belgium, the UK, the Netherlands and Ireland. It provided an opportunity to explore practical suggestions to improve health equity within primary care. An introductory presentation on the subject of health equity was given by a team led by Professor Sara Willems. As a group, we discussed each of the following questions in turn and simultaneously recorded the key points volunteered by individual participants onto whiteboards.

1. What are the barriers and facilitators to addressing SDH at the practice level?
2. What can be done in the consultation to improve health equity?
3. Can you think of good examples of training for primary care practitioners that address heath equity?
4. Should all primary care practitioners receive training about SDH? And if so, what and how much training?

The facilitators encouraged contributions from all workshop participants. This report gives a summary of the key points raised by the workshop participants.

Before leaving the workshop participants were also asked to complete a survey consisting of fourteen questions about different inequity-reducing activities. They were asked to rate the priority of each activity on a scale of 1 to 5 (1 = not a current priority, 5 = top priority). Ten completed surveys were returned. For each health inequity-reducing activity, scores were lower for the current state compared to what should ideally be the case. For example, one health inequity-reducing activity was ‘Reform of medical education to incorporate health equity and cultural competency training’. Participants gave a mean score of 2.11 (+/−SD 0.99) for current priority compared to 4.6 (+/−SD 0.52) for the ideal priority reflecting the difference between what is and what should be happening with regard to the incorporation of health equity issues in medical education.

### Barriers to addressing social determinants of health at the practice level

There were several barriers suggested by workshop participants, which will be considered in turn. First, poor health literacy was considered a significant obstacle. Health literacy is defined as “the degree to which individuals have the capacity to obtain, process and understand basic health information and services needed to make appropriate health decisions” [6]. In other words, there are many elements needed for a patient to be health literate; including cultural knowledge, listening, reading and writing skills, as well as numeracy.

Austerity measures and other sequelae of the global financial crisis of 2007/8 were also mentioned as barriers to addressing health inequity. In 2012, the “GPs at the Deep End” group (www.gla.ac.uk/deepend), representing the hundred most deprived practices in Scotland, published a report detailing the impact of austerity measures on patients [7]. The report described the direct and indirect effects of cuts to benefits and services, including deteriorating physical and mental health problems for patients, and increased stress and workload for primary care practices. The Deep End group have subsequently lobbied the Scottish Government, resulting in a parliamentary debate over funding and resources in deprived areas. Although the profile of this important issue has been raised, substantive increases to primary care resources have yet to be made. A recent publication illustrated that general practice funding in Scotland does not match clinical need [8]. This group is a great example of GPs coordinating action and advocacy with strength in numbers, to address the inequities directly affecting their patients. Groups modelled on this initiative in Scotland are now being established by GPs working in areas of deprivation in other areas of the UK, Ireland and Australia.

Having inadequate time for GP consultations was another barrier mentioned by participants. Research has shown that GP consultation length varies across Europe and even within individual countries, from 7.8 min per appointment in Spain to 15.6 min in Switzerland [9]. The pressures of time constraints and ever-increasing health complexity leave little time for the GP to consider and address the wider social context and SDH.

### Facilitators/suggestions for change at the practice level

Some participants suggested that more effort should be made to involve frontline GPs, particularly those with experience of working in deprived areas, in creating health policies. One example of where this has been done is in England, where GPs now sit on local clinical commissioning groups which allocate funding and resources for health services. It remains to be seen, however, what the health equity impact of these primary care reforms will be. Another example is the Partnership for Health Equity in Ireland (www.healthequity.ie), which has been established to allow GPs, researchers, educators, health planners and policy makers to formally collaborate on a number of initiatives to improve the health of marginalised groups and those living in deprived areas.

Participants also suggested making better use of the time and skills of other healthcare professionals, such as pharmacists and practice nurses, to engage with patients on health issues, thereby freeing up more time for GPs to deal with more complex medical problems and care coordination. One example of this came from Belgium, where administrative staff in pilot practices were involved in the education of vulnerable patients to improve their health literacy.

Longer consultation times to deal with more complex cases and reducing the number of patients on a GP’s total list (to improve relationships and continuity) were further suggestions. Longer consultations can only be achieved, however, by reducing the number of available appointments or increasing the GP workforce. The development of trusting relationships between GPs and patients was another facilitator raised at the workshop. A recent Royal College of General Practitioners report on health inequalities in the UK suggests incentivising ways of working which promote continuity, particularly in areas with high multimorbidity [10].

Having good access to trained interpreters for patients was suggested at the workshop. Using interpreters requires additional time and resource for practices which are already over-stretched. Once again, patients and practices in deprived areas are most affected, as they are more likely to have immigrant and asylum seeker populations. Unfortunately, the provision of interpreters can be limited and underfunded. Improved resources and increased consultation time would help with this issue.

### Suggestions for improved awareness of health inequities at a training level

The importance of covering health inequities in medical education was raised as a priority by workshop participants, repeating the conclusions of the WONCA 2013 Workshop [5]. Recent research by Williamson et al. highlighted the need for concerted effort to educate students on tackling health inequalities [11]. Suggestions for core learning included knowing the evidence base for health inequality aspects of common chronic conditions, learning specific communication skills and the acknowledgement of prejudices harboured against patients from different backgrounds. Another example is an initiative from Baltimore in the USA that promotes the teaching of social justice and collaboration with non-governmental organisations as part of their curriculum for working with marginalised groups [12].

The Istanbul workshop participants suggested allowing students to observe a patient or family from a deprived area over time and also encouraging contact with marginalised groups out with the medical setting. Published research has echoed these sentiments with suggestions that knowledge of the ‘lived experience’ of patients at the margins of society and the adversity they face are crucial skills for the development of conscientious GPs of the future [13]. It was also acknowledged that to incentivise and encourage students to learn about these issues, formal assessment of these topics would be required.

## Conclusions

The health equity workshop at the 2015 WONCA Europe conference continued the work of the WONCA 2013 workshop by providing an excellent opportunity to further explore the barriers and facilitators for addressing the social determinants of health in primary care with participants of varying levels of experience from a wide variety of countries. However, as Julian Tudor Hart, who coined the term ‘inverse care law’, said: intellectual opposition to injustice is only the beginning of social understanding [2]. The next step is to turn words into deeds, and for that we need the required evidence as well as the support of both politicians and the public. Of note, the International Journal for Equity in Health has recently published a thematic series of various interventions worldwide designed to improve equity in health [14]. Examples such as these are crucial for shaping future equitable health care.

For further information on the WONCA Special Interest Group on Health Equity see www.globalfamilydoctor.com/groups/SpecialInterestGroups/HealthEquity.aspx.

## Abbreviations

GP, general practitioner; SDH, Social Determinants of Health; WONCA, World Organization of Family Doctors
